# Interactions of Linalool and Linalyl Acetate with Selected Dog Cytochrome P450 (CYP) Proteins Identified by In Silico Drug Discovery Followed by Molecular Docking Analysis

**DOI:** 10.3390/ph18101499

**Published:** 2025-10-06

**Authors:** Raquel Rodrigues Soares-Santos, Arun Kumar Jaiswal, Renata Cristina Mendes Ferreira, Vasco Ariston de Carvalho Azevedo, Flávia Figueira Aburjaile, Benito Soto-Blanco

**Affiliations:** 1 Laboratory of Veterinary Toxicology, Department of Veterinary Clinics and Surgery, School of Veterinary Medicine, Universidade Federal de Minas Gerais, Belo Horizonte 31270-901, MG, Brazil; qlrodrigues@gmail.com; 2 Laboratory of Cellular and Molecular Genetics, Institute of Biological Sciences, Universidade Federal de Minas Gerais, Belo Horizonte 31270-901, MG, Brazil; arunjaiswal1411@gmail.com (A.K.J.); vascoariston@gmail.com (V.A.d.C.A.); 3 Department of Physiology and Biophysics, Institute of Biological Sciences, Universidade Federal de Minas Gerais, Belo Horizonte 31270-901, MG, Brazil; recmferreira@gmail.com; 4 Department of Preventive Veterinary Medicine, School of Veterinary Medicine, Universidade Federal de Minas Gerais, Belo Horizonte 31270-901, MG, Brazil; faburjaile@gmail.com

**Keywords:** molecular docking, *Lavandula angustifolia*, metabolism, CYP, linalool, linalyl acetate, dogs

## Abstract

**Background:** Cytochrome P450 (CYP450) enzymes play a central role in the metabolism of xenobiotics, including plant-derived compounds such as terpenoids. **Objectives**: This study aimed to predict the molecular interactions of linalool (LIN) and linalyl acetate (LINAct), major constituents of lavender essential oil, with the canine CYP2B11, CYP2C21, and CYP2D15 isoforms, using in silico approaches. **Methods**: Three-dimensional (3D) models of the target enzymes were generated through homology modeling using SWISS-MODEL and validated based on global model quality estimate (GMQE) and QMEAN Z-score metrics. Ligand structures were optimized in the Molecular Operating Environment (MOE), and pharmacophoric features were analyzed. Molecular docking simulations were performed using AutoDock Vina, followed by visualization of interactions in MOE. **Results**: LIN and LINAct exhibit favorable binding affinities with all three isoforms, suggesting their potential as substrates or modulators. Hydrogen bonding and hydrophobic interactions were the predominant forces stabilizing the ligand–enzyme complexes. **Conclusions**: These findings provide a computational basis for understanding the hepatic metabolism of LIN and LINAct in dogs, offering preliminary insights into the role of specific CYP isoforms in their biotransformation.

## 1. Introduction

The rapid advancement of computational technologies has transformed the landscape of drug discovery and development, positioning bioinformatics at the forefront of modern biomedical research [1]. The integration of bioinformatics into drug discovery and development has revolutionized the way we understand and manipulate biological systems for therapeutic benefit [2]. As a multidisciplinary field, bioinformatics combines computational tools and biological data to accelerate drug development, enabling researchers to identify targets, simulate molecular interactions, and predict pharmacological outcomes with unprecedented accuracy [3]. By integrating biology, computer science, mathematics, and statistics, bioinformatics facilitates the analysis and interpretation of vast biological datasets, enabling researchers to uncover meaningful insights into disease mechanisms, identify potential drug targets, and predict therapeutic responses [1]. In a field where traditional drug development is often expensive, time-consuming, and prone to high failure rates, the incorporation of in silico approaches such as molecular docking, molecular dynamics simulations, and computer-simulated experiments has emerged as a powerful and cost-effective alternative [4]. In silico drug discovery, such as molecular docking, leverages bioinformatics tools for tasks such as target identification, target preparation, molecular docking, virtual screening, and ADMET (absorption, distribution, metabolism, excretion, and toxicity) profiling [5]. One of the key components of this transformation is the use of in silico methods, which utilize computer-based simulations to reduce the need for time-consuming and costly laboratory and animal testing in the early phases of research. These approaches offer critical advantages, such as high-throughput screening of compounds, detailed structural analyses of drug–target interactions, and early toxicity prediction, which contribute to more efficient and rational drug design [5].

**Figure 1:**
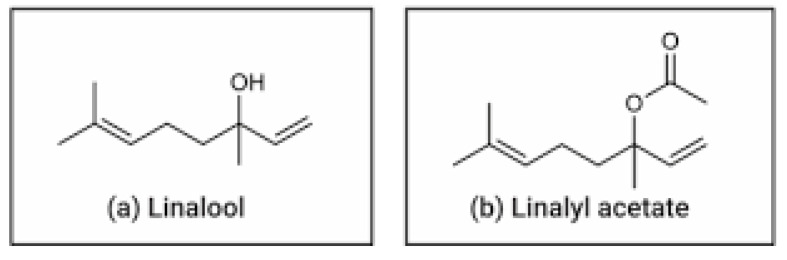
Chemical structures of (**a**) linalool and (**b**) linalyl acetate.

Among the vast array of natural products being explored for therapeutic use, essential oils have garnered significant attention due to their unique essence and complexity of bioactive compounds, which exhibit antimicrobial, anti-inflammatory, anxiolytic, and antioxidant properties [6]. Essential oils are part of the plant’s defense system and can be extracted from various parts of the plant, including flowers, seeds, leaves, fruits, wood, and roots. Essential oils derived from aromatic plants are rich in terpenes, phenols, and ester compounds, which are known for their diverse pharmacological activities [6]. Lavender (*Lavandula angustifolia*) is a well-known essential oil with a long history of use in traditional medicine and modern aromatherapy, and the chemical composition of LEO is responsible for its aroma, floral notes, and therapeutic action. Lavender oil contains key constituents such as linalool and linalyl acetate (Figure 1), the concentration of which determines the quality of the essential oil, as well as its therapeutic properties [7] ranging from anxiolytic and sedative properties to wound healing and antimicrobial action. Due to these effects, lavender is increasingly being incorporated into complementary and integrative medicine practices for both humans and animals [8]. It is important to emphasize that, although there is common sense in conferring pharmacological safety to natural products [9], some plant compounds can be potentially toxic to dogs, like theobromine [10], caffeine, and certain essential oils, such as tea tree [11,12], eucalyptus, and pennyroyal [13].

Despite its growing use in veterinary applications, especially in canine health, there is limited knowledge regarding the metabolism of lavender compounds in dogs [14]. Understanding how these bioactive compounds are absorbed, distributed, metabolized, and excreted in canine physiology is essential for evaluating their safety, efficacy, and dosage. However, ethical, logistical, and financial constraints often limit the extent of in vivo testing in animals. This gap highlights the need for in silico studies that can simulate metabolic pathways, predict interactions with canine enzymes (e.g., cytochrome P450 family), and assess potential toxicity or bioavailability risks.

In this work, we employed in silico drug discovery, followed by molecular docking analysis, which was performed with selected cytochrome P450 (CYP) proteins, CYP2B11, CYP2C21, and CYP2D15, of the dog against linalool and linalyl acetate. The strength of molecular docking analysis in this context lies in its ability to generate clinically relevant predictions about the pharmacokinetics and dynamics of lavender-derived compounds (linalool, linalyl acetate) in dogs. By leveraging molecular docking and molecular dynamics simulation, as well as ADMET profiling, we can predict how these compounds behave in canine systems. This prediction not only informs safer formulation and dosing strategies but also contributes to the broader field of veterinary pharmacology. Ultimately, via the application of in silico drug discovery, followed by molecular docking analysis of CYP proteins CYP2B11, CYP2C21, and CYP2D15 of dogs against the ligand molecules linalool and linalyl acetate from lavender essential oil, their pharmacophore properties and binding sites were identified, which may provide a foundation for more targeted and effective therapeutic interventions. This study aimed to predict the molecular interactions of linalool (LIN) and linalyl acetate (LINAct), major constituents of lavender essential oil, with the canine CYP2B11, CYP2C21, and CYP2D15 isoforms, using in silico approaches.

## 2. Results

### 2.1. Molecular Modeling of the Target Proteins CYP2B11, CYP2C21, and CYP2D15

**Figure 2:**
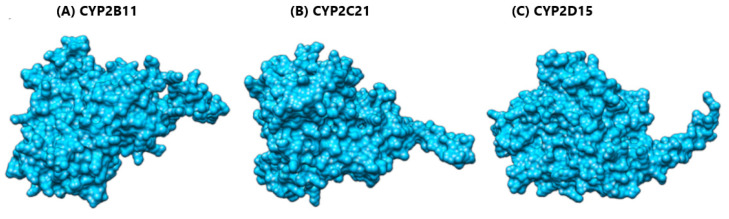
Modeled 3D structure of canine proteins CYP2B11, CYP2C21, and CYP2D15 from the SWISS-MODEL server against: (**A**) template A0A836CX50.1.A (Cytochrome P450 of *Ovis aries* (Sheep)). GMQE = 0.94. (**B**) A0A6P3GP28.1.A (Cytochrome P450 of *Bison bison* (North American plains bison)). GMQE = 0.94, and (**C**) E3VVY0.1.A (Cytochrome P450 of *Panthera tigris altaica* (Siberian tiger)). GMQE = 0.93, respectively.

**Figure 3:**
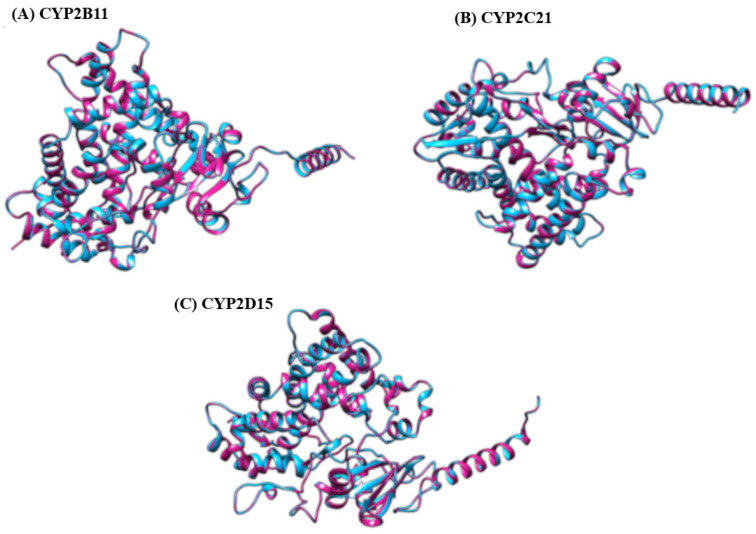
Superimposed structure of: (**A**) modeled protein CYP2B11 (blue) with template A0A836CX50.1.A (Cytochrome P450 of *Ovis aries* (Sheep)) (red). GMQE = 0.94, (**B**) Superimposed structure of modeled protein CYP2C21 (blue) with template A0A6P3GP28.1.A (Cytochrome P450 of North American plains bison (Bison bison) (red)). GMQE = 0.94, and (**C**) protein CYP2D15 (blue) with template E3VVY0.1.A (Cytochrome P450 of *Panthera tigris altaica* (*Siberian tiger*)) in red). GMQE = 0.93.

We used the SWISS-MODEL server to perform homology modeling of our protein targets. The server identified templates against the CYP2B11, CYP2C21, and CYP2D15 proteins from AlphaFoldDB to generate the 3D structure of our proteins (Figure 2), followed by the further generation of superimposed structures (Figure 3). For CYP2B11, the best template structure was A0A836CX50.1.A (cytochrome P450 of *Ovis aries* (sheep)) and showed sequence identity 81.30 and sequence similarity 0.56 with the template structure. For protein CYP2C21, the best template structure identified was A0A6P3GP28.1.A (cytochrome P450 of *Bison bison* (North American plains bison)) and showed sequence identity 79.18 and sequence similarity 0.55 with the template structure. For the protein CYP2D15, the best identified template was E3VVY0.1.A (cytochrome P450 of *Panthera tigris altaica* (Siberian tiger)) and showed a sequence identity of 80.20 and sequence similarity of 0.56 with the template structure.

**Figure 4:**
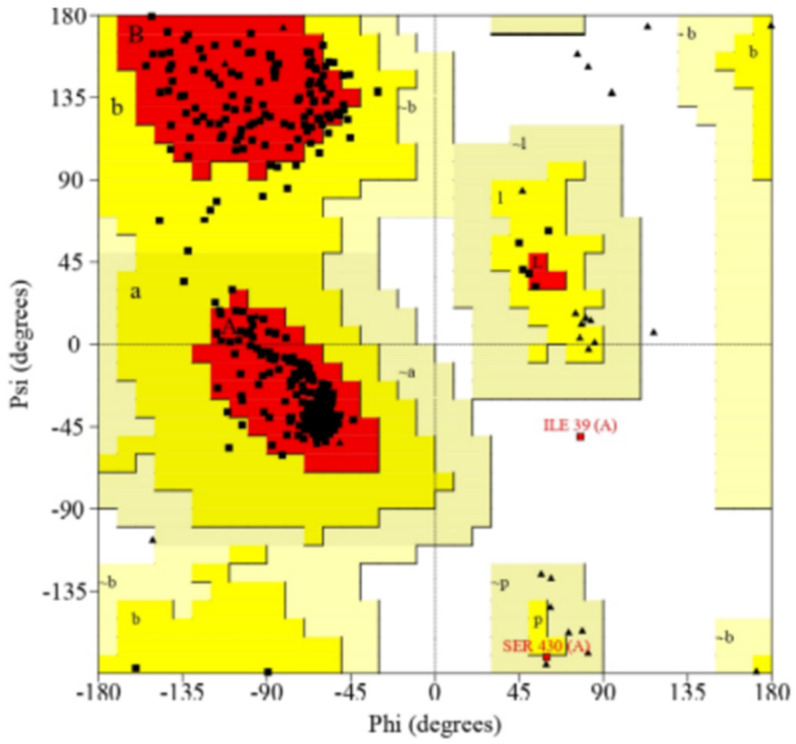
Structure validation Ramachandran graph of modeled CYP2B11 protein from Procheck SAVES server.

**Figure 5:**
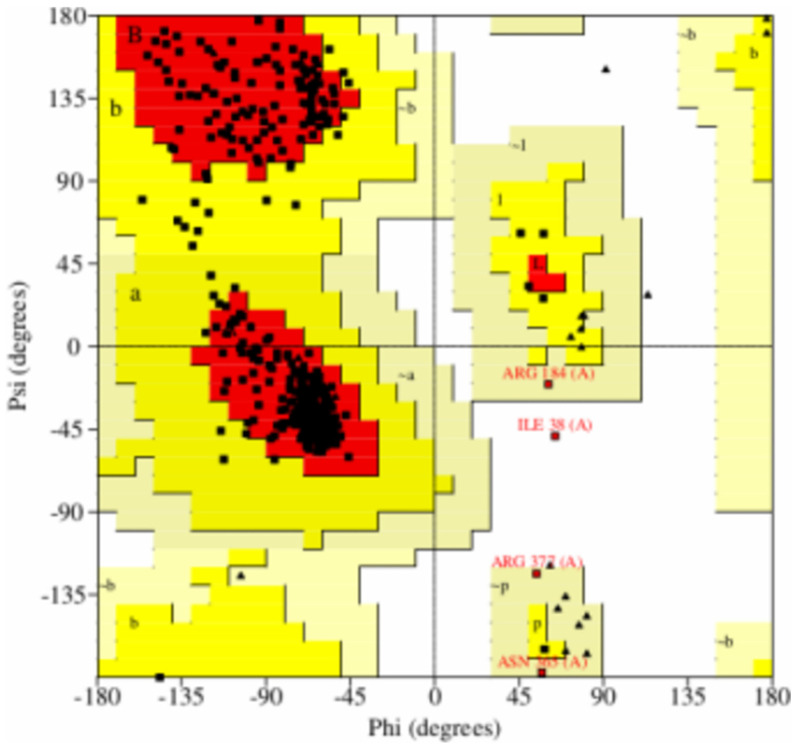
Structure validation Ramachandran graph of modeled CYP2C21 protein from Procheck SAVES server.

**Figure 6:**
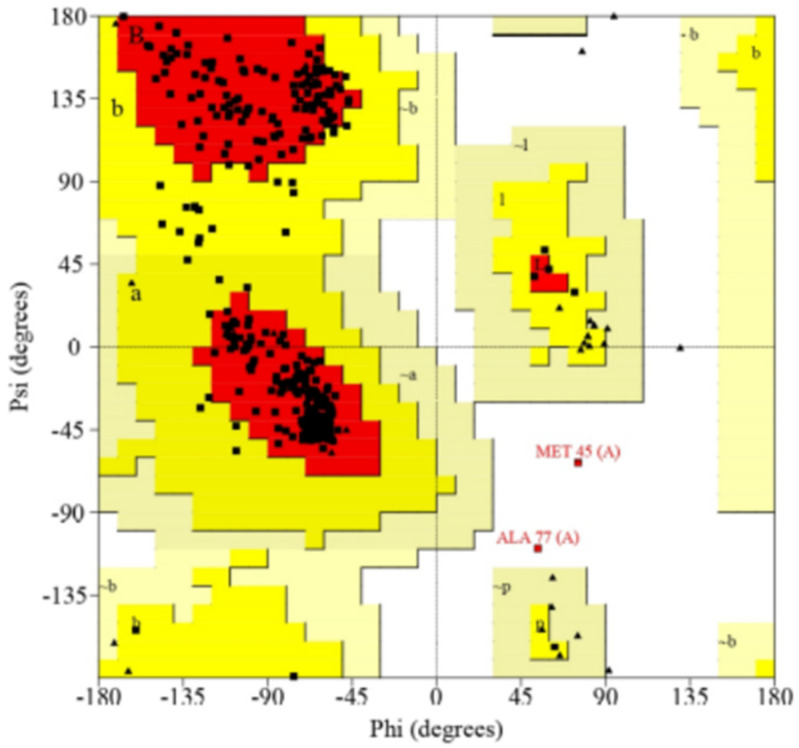
Structure validation Ramachandran graph of modeled CYP2D15 protein from Procheck SAVES server.

After generating the 3D structure of proteins, we further performed structure validation analysis to generate the Ramachandran graph from the Procheck SAVES server and identified the modeled protein structure of CYP2B11, which showed that 92.5% residues were in most of the favored regions. The results were as follows: residues in additional allowed regions (a, b, l, p): 30 (7.0%); residues in generously allowed regions (−a, −b, −l, −p): 1 (0.2%); residues in disallowed regions: 1 (0.2%); number of non-glycine and non-proline residues: 426; number of end-residues (except Gly and Pro): 1; number of glycine residues (shown as triangles): 34; number of proline residues: 31; total number of residues: 492. As demonstrated in Figure 4, the modeled protein structure of CYP2C21 showed that 92.1% residues were in most of the favored regions; residues in additional allowed regions (a, b, l, p): 30 (6.9%); residues in generously allowed regions (−a, −b, −l, −p): 3 (0.7%); residues in disallowed regions: 1 (0.2%); number of non-glycine and non-proline residues: 432; number of end-residues (except Gly and Pro): 2; number of glycine residues (shown as triangles): 27; number of proline residues: 29; total number of residues: 490. As shown in Figure 5, the modeled protein structure of CYP2D15 showed that 92.1% residues were in most favored regions. The results were as follows: residues in additional allowed regions (a, b, l, p): 32 (7.5%); residues in generously allowed regions (−a, −b, −l, −p): 0 (0.0%); residues in disallowed regions: 2 (0.5%); number of non-glycine and non-proline residues: 428; number of end-residues (except Gly and Pro): 2; number of glycine residues (shown as triangles): 33; number of proline residues: 37; total number of residues: 500. Figure 6 shows that our modeled protein structure is of good quality.

### 2.2. Molecular Docking Analysis

No experimental data were manually manipulated in this study. The results presented were based on computational predictions made using MOE’s internal algorithms without modifying the tool’s default parameterization.

Molecular docking analysis was performed using AutoDock Vina to identify the binding energy between the modeled protein targets and the ligand compounds linalool and linalyl acetate.

**Figure 7:**
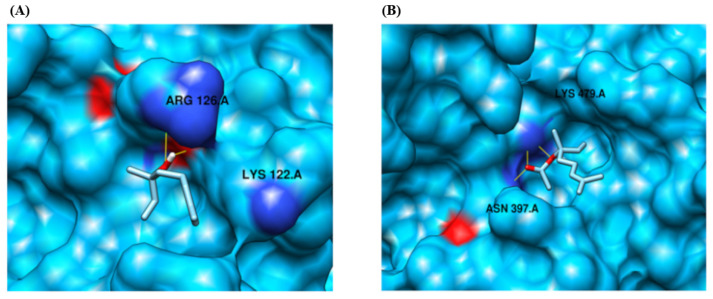
Molecular docking analysis of the CYP2B11 protein using the MOE tool. (**A**) Binding interaction with the ligand compound linalool. Represented by a yellow line, a hydrogen bond between the molecule and the residue ARG.A. (**B**) Binding interaction with the ligand compound linalyl acetate. A yellow line represents a hydrogen bond between the molecule and residue ASN 397.A. CYP2B11 is represented in deep sky blue. The electronegative zone of the protein is indicated in red. In purple, amino acids with potential interactions with each molecule are represented. ARG 126.A = arginine; LYS 122.A = lysine.

**Figure 8:**
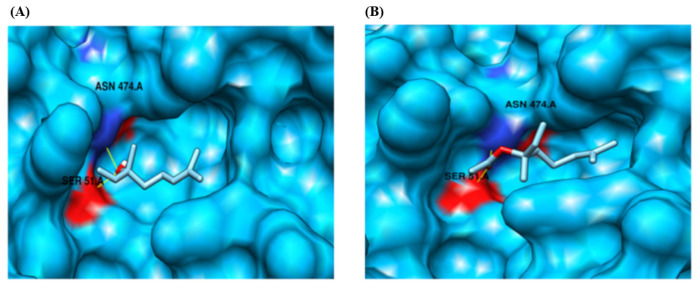
Molecular docking analysis of the CYP2C21 protein using the MOE tool. (**A**) Binding interaction with the ligand compound linalool. Represented by a yellow line, a hydrogen bond between the molecule and the residue SER 51.A. and ASN 474.A. (**B**) Binding interaction with the ligand compound linalyl acetate. Represented by a yellow line, a hydrogen bond between the molecule and residue SER 51.A and ASN 474.A. CYP2C21 is represented in deep sky blue. The electronegative zone of the protein is indicated in red. In purple, amino acids with potential interactions with each molecule are represented. SER 51.A = serine; ASN 474.A = asparagine.

**Figure 9:**
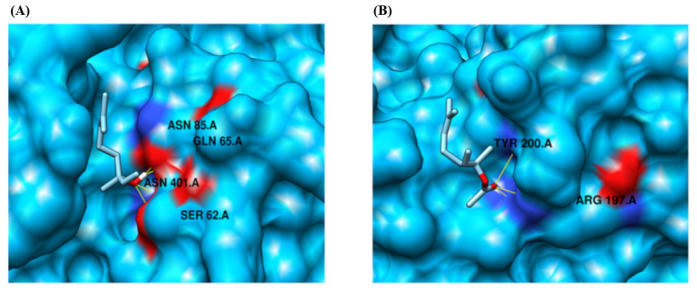
Molecular docking analysis of the CYP2D15 protein using the MOE tool. (**A**) Binding interaction with the ligand compound linalool. Represented by a yellow line, a hydrogen bond between the molecule and the residue ASN 401.A. and SER 62.A. (**B**) Binding interaction with the ligand compound linalyl acetate. Represented by a yellow line, a hydrogen bond between the molecule and residue TYR 200.A and ASN 474.A. CYP2D15 is represented in deep sky blue. The electronegative zone of the protein is indicated in red. In purple, amino acids with potential interactions with each molecule are represented. ASN 401.A = asparagine; SER 62.A = serine; TYR 200.A; = tyrosine; GLN 65.A = glutamine; ASN 85.A = asparagine; ARG 197.A = arginine.

Based on our docking analysis, we identified that the protein target CYP2B11 binds to residues LYS 122 and ARG 126, forming a hydrogen bond with the ligand compound linalool (Figure 7, Table 1). The protein also exhibited a binding interaction and formed hydrogen bonds with residues ASN 397 and LYS 479 in response to the ligand compound linalyl acetate. Similarly, linalool and linalyl acetate were docked against the CYP2C21 protein (Figure 8, Table 1). Based on our docking analysis, we identified that CYP2C21 showed a binding interaction with residues ASN 474 and SER 51, forming hydrogen bonds with linalool and linalyl acetate, respectively. Protein CYP2D15 showed a binding interaction and formed a hydrogen bond with residues SER 62, GLN 65, ASN 85, and ASN 401 against ligand compound linalool (Figure 9, Table 1). Additionally, protein CYP2D15 showed interaction with residues ARG 197 and TYR 200, forming a hydrogen bond against the ligand compound linalyl acetate. The Chimera visualization software was used to visualize the binding between proteins and ligand compounds and extract the binding images.

### 2.3. Ligand-Based Drug Design

#### 2.3.1. Pharmacophore Properties of Linalool and Linalyl Acetate

**Figure 10:**
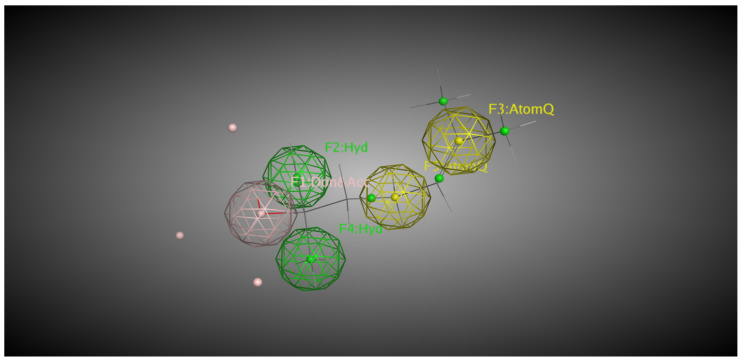
Mapping of the pharmacophoric properties of linalool using the MOE tool. The molecule has two hydrophobic groups, represented in green (F2:Hyd and F4:Hyd), and one hydrogen bond donor region, represented in pink (F1:DonAcc). Two AtomQ are observed in yellow. F = feature number; Hyd = Hydrophobic region; DonAcc = Dual donor/acceptor; AtomQ = Qualifying Atom.

**Figure 11:**
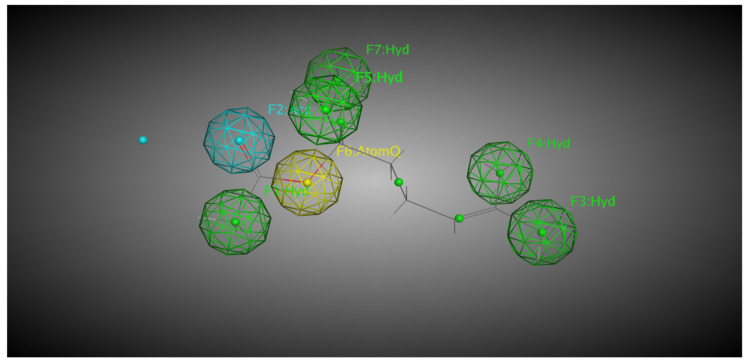
Mapping of the pharmacophoric properties of linalyl acetate using the MOE tool. The molecule has five hydrophobic groups, represented in green (F1, F3, F4, F5, and F7:Hyd) and one hydrogen bond acceptor region, represented in blue (F2:Acc). AtomQ is observed in yellow. F = feature number; Hyd = Hydrophobic region; Acc = Dual Hydrogen bond acceptor; AtomQ = Qualifying Atom.

Both linalool and linalyl acetate were evaluated for their pharmacophoric characteristics using MOE software (v. 2024.0601). For linalool, five pharmacophoric characteristics were identified, including two hydrophobic groups located in the terpene side chain, a dual region (i.e., hydrogen bond acceptor or donor) located in one of the terminal zones of the molecule, and two atoms in the other terminal zone (Figure 10). AtomQ is a generic pharmacophoric region that does not fit as a hydrogen-bond donor or acceptor but is relevant for interactions with biological targets. Linalyl acetate, on the other hand, has very different pharmacophoric characteristics. Five hydrophobic regions distributed throughout the terpene structure, one hydrogen-bond acceptor region located at the opposite end to the terpene nucleus, and one AtomQ between the acceptor and hydrophobic regions were identified, totaling seven pharmacophoric characteristics (Figure 11).

#### 2.3.2. Molecular Docking

**Figure 12:**
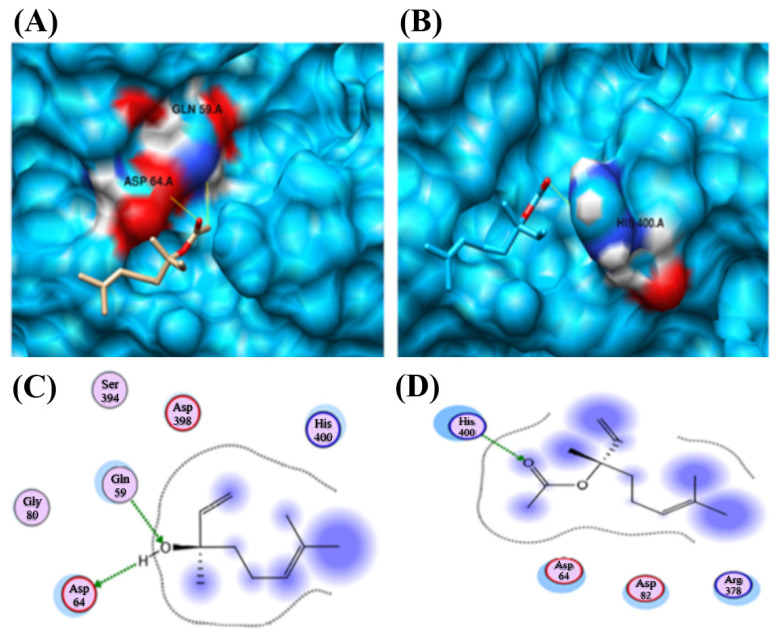
Molecular docking analysis and binding map between linalool, linalyl acetate, and CYP2B11 using the MOE tool. (**A**) 3D representation of the molecular docking between linalool and CYP2B11. The yellow lines indicate that the ligand interacts with hydrogen bonds with the amino acids GLY (glycine) and ASP (asparagine). (**B**) 3D representation of molecular docking between linalyl acetate and CYP2B11. The yellow lines indicate that the ligand has hydrogen interactions with the amino acid HIS 400 (histidine). (**C**) 2D representation of the binding map between linalool and the main amino acids involved in the interactions between the compound and CYP2B11. (**D**) 2D representation of the bond map between linalyl acetate and the main amino acids involved in the interactions between the compound and CYP2B11. The red color represents the electronegative regions in the 3D representation. Blue represents positive regions in the 3D representation. In the 2D representation, the green dotted line represents hydrogen bonds, red halos represent possible hydrogen bonds, and blue halos represent nonpolar interactions. ARG = Arginine; ASP = Aspartic acid; GLN = Glutamine; SER = Serine.

Docking was performed for CYP2B11 against linalool and linalyl acetate. Linalool shows promising results against CYP2B11. The complete docking results are shown in Table 2. In the case of linalool, two interactions were observed. The predicted hydrogen bond donor and acceptor features of linalool were found to interact with GLN 59 and ASP 64 residues of the CYP2B11 protein. However, in the case of linalyl acetate, the predicted hydrogen-bond acceptor feature was observed to interact with HIS 400 residues of the CYP2B11 protein (Figure 12).

**Figure 13:**
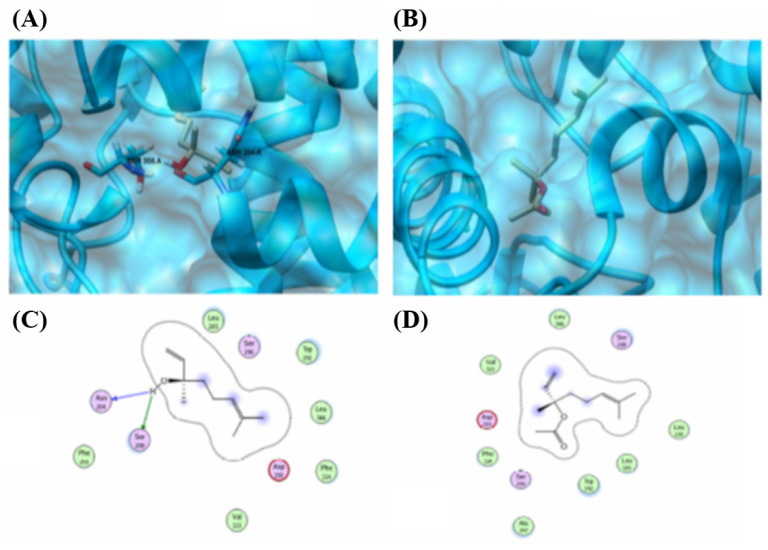
Molecular docking analysis and binding map between linalool, linalyl acetate, and CYP2C21 using the MOE tool. (**A**) 3D representation of the molecular docking between linalool and CYP2C21. The yellow lines indicate that the ligand interacts with hydrogen bonds with the amino acids SER (serine) and ASN (asparagine). (**B**) 3D representation of molecular docking between linalyl acetate and CYP2C21. The absence of yellow lines indicates that there are no hydrogen interactions with the protein. (**C**) 2D representation of the binding map between linalool and the main amino acids involved in the interactions between the compound and CYP2C21. (**D**) 2D representation of the bond map between linalyl acetate and the main amino acids involved in the interactions between the compound and CYP2C21. Blue represents positive regions in the 3D representation (50% transparency). In the 2D representation, the green dotted line represents hydrogen bonds, and the blue dotted line represents a weak hydrogen bond. Red halos represent possible hydrogen bonds. ASP = Aspartic acid; GLU = Glutamic acid; ILE = Isoleucine; LEU = Leucine; PHE = Phenylalanine; THR = Threonine; TYR = Tyrosine; VAL = Valine.

**Figure 14:**
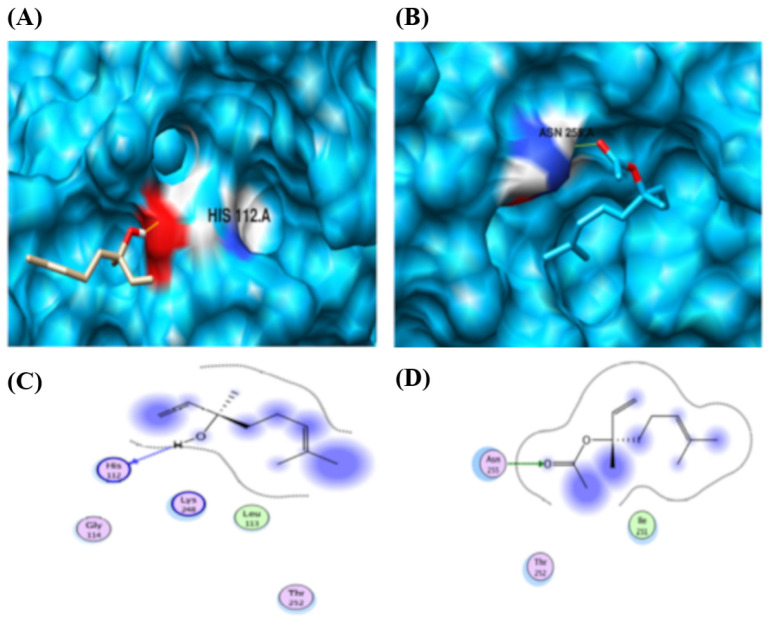
Molecular docking analysis and binding map between linalool, linalyl acetate, and CYP2D15 using the MOE tool. (**A**) 3D representation of the molecular docking between linalool and CYP2D15. The yellow lines indicate that the ligand interacts with hydrogen bonds through the amino acid histidine (HIS). (**B**) 3D representation of molecular docking between linalyl acetate and CYP2D15. (**C**) 2D representation of the binding map between linalool and the main amino acids involved in the interactions between the compound and CYP2D15. The yellow lines indicate that the ligand has hydrogen interactions with the amino acid ASN (asparagine). (**D**) 2D representation of the bond map between linalyl acetate and the main amino acids involved in the interactions between the compound and CYP2D15. The red color represents the electronegative regions in the 3D representation. Blue represents positive regions in the 3D representation. In the 2D representation, the green dotted line represents hydrogen bonds, and the blue dotted line represents a weak hydrogen bond. Blue halos represent nonpolar interactions. GLY = Glycine; ILE = Isoleucine; LEU = Leucine; LYS = Lysine; THR = Threonine.

Similarly, linalool and linalyl acetate were docked against the CYP2C21 protein. (Figure 13, Table 2). Linalool exhibited two interactions with the CYP2C21 protein. The same features of linalool were observed to contribute to interactions (Figure 14A,C). Interactions were observed between the compound and ASN 204 and SER 208. However, linalyl acetate showed no interaction with CYP2C21 protein.

Linalool and linalyl acetate were docked against the CYP2D15 protein (Table 2). Both ligands exhibited a single interaction with the CYP2D15 protein. Interestingly, the same features of both ligands were observed to contribute to interactions (Figure 14). Linalool interacts with HIS 112, and linalyl acetate interacts with residue ASN 255.

## 3. Discussion

This in silico study was conducted to gather initial data on the interactions between canine CYP enzymes and the compounds studied. The SWISS-MODEL tool was used to perform 3D modeling of the CYP enzymes, offering accessibility and ease in modeling protein complexes. The modeled target of CYP2B11 and CYP2C21 had a GMQE of 0.94, whereas that of CYP2D15 had a GMQE of 0.93. These values indicate that the models were reliable and structurally consistent with known CYP450, as they were close to the maximum GMQE value, making them suitable targets for subsequent analysis. Importantly, these scores exceeded the thresholds typically considered acceptable for comparative modeling, suggesting that the predicted structures are suitable for exploring ligand interactions. Nevertheless, it must be emphasized that such models do not fully reproduce the conformational flexibility or heme-dependent features of CYP enzymes, which should be addressed in future refinements.

Structural characterization of a template protein is a prerequisite for homology modeling, which requires a sufficient sequence similarity to the target protein. If a suitable structure does not exist, alternative modeling approaches, such as ab initio or de novo methods, need to be employed [15,16,17]. This stage of in silico analysis constructs a 3D model of the target protein using a comparable model, based on the principle that proteins with similar structures often display analogous biological functions [16,18,19]. In this study, the structures of the CYP2B11, CYP2C21, and CYP2D15 enzymes were constructed from pre-existing targets of CYP450 from sheep (*Ovis aries*), North American plains bison (*Bison bison*), and Siberian tiger (*Panthera tigris altaica*), respectively. Although there are small differences between mammalian CYPs, studies have shown that the catalytic sites of these enzymes are generally the same [20,21] This supports the use of CYPs from other species as models for studying the interactions of molecules with canine CYPs. Figure 4, Figure 5 and Figure 6 show that the residues of the three enzymes are generally overlapping, indicating a high similarity between the target protein and the model created by SWISS-MODEL. Furthermore, the predominance of green color in these figures indicates that the similarity is greater than the differences between the target and model. These results align with well-established models studied by other researchers, showing that CYP modeling can overlap across species. For example, Mestres [22] demonstrated that it is feasible to model CYPs of the 2A superfamily with good quality from other species, since the structural similarity is high (RMSD < 2Å). Furthermore, Szklarz et al. [23] showed that even when using bacterial templates to model mammalian CYPs, these models can be useful for studying substrate-enzyme interactions. Although experimental crystallographic structures of canine CYPs are currently unavailable, the constructed models successfully replicate essential structural features necessary for docking. This underscores the efficacy of our approach as an initial step in predicting the metabolic risks associated with essential oil components in veterinary medicine.

The percentage of identity between the target structure and the model created was also determined. The proposed reference values for the percentage identity between the modeled structure and the target are variable. According to Lohning et al. [16], a sequence must have more than 35% homology to be considered a reliable model, while other authors establish that the value must exceed 50% [16,24]. In general, CYP enzymes are recognized for their robust structural nature, and reliable homology models can often be generated even at lower identity values. Mestres [22] demonstrated that even within CYP families exhibiting significant sequence divergence (10–27%), still maintain remarkable structural conservation, particularly in the active region. Similarly, Werck-Reichhart and Feyereisen [25] emphasized that despite low sequence identity (<20%), the overall structural configuration of the P450 family, especially around the heme, remains highly conserved. Thus, the model created for each CYP under study is reliable, as it maintains an identity of at least 79.18%.

The Ramachandran plot is a valuable tool for validating the model created by SWISS-MODEL. If more than 90% of the residues are in favorable regions, the 3D protein model can be considered stereochemically reliable for molecular docking [26]. For the three CYPs modeled, the percentage of residues in favorable regions was greater than 92%, corroborating the other data [27,28] on the reliability of the obtained 3D structures. Although Ramachandran is a good stereochemical metric, it does not guarantee accuracy in the active site or the protein dynamics.

In silico docking indicated that linalool forms stable interactions with CYP2B11 through hydrogen bonds with Ser and Thr residues, with higher predicted affinity compared to CYP2C21 and CYP2D15. Previous studies on liver microsomes have demonstrated that monoterpenes, such as linalool, are preferentially metabolized by CYP enzymes [29,30,31], supporting our prediction of selective binding. Molecular docking enables the prediction of interactions between two molecules, specifically a protein structure (the target protein) and a ligand (a compound molecule or structure). It enables the assessment of the structure-ligand complex and its relationship to biological systems, thereby enhancing the capacity to identify ligands with higher affinity for the target protein [16,17,32]. The residues that participate in molecular docking have chemical features that are crucial for effective biochemical interactions. Charged residues (basic or acidic) have a positive charge and favor hydrogen bonds. Thus, in electropositive or polar environments, the interaction tends to be stronger, especially with electron-donating or electron-accepting atoms. These residues tend to perform initial ligand recognition in a more dynamic, i.e., less stable manner. Neutral residues do not possess any electrical charge. They form strong hydrogen bonds, which favor more stable and directed interactions. The result is the formation of more stable complexes. Aromatic residues form hydrogen bonds with the hydroxyl group (-OH) and hydrophobic interactions if the ligand contains an aromatic ring in its structure. This characteristic imparts a versatile complex but is highly dependent on the orientation of the ligand [33,34,35]. Although docking predicts favorable interactions, the absence of protein dynamics and solvent effects may result in an overestimation of binding stability. Furthermore, the conformation of the modeled residues may deviate slightly from those of native enzymes, potentially affecting in vitro metabolic processes. These findings provide a sound rationale for selecting these CYP as targets in experimental investigations of linalool metabolism.

The docking analysis of CYP2B11 using the MOE tool showed that both LIN and LINA are capable of interacting through hydrogen bonds with the residues ARG 126.A, LYS 122.A, and ASN 397.A, in addition to electrostatic interactions between these residues. These characteristics and the positions of the residues favor the stable docking of the protein [17,32], suggesting that CYP2B11 can metabolize LIN and LINA. In vitro studies with human microsomes have shown that the CYP2B subfamily can metabolize other terpenes, such as (-)-verbenone [36] and (-)-fenchone [37]. Some monoterpenes, such as citral and geraniol, specifically interact with CYP2B6, the ortholog of canine CYP2B11, and moderately inhibit it [38].

By comparing the interactions between the terpenoids and the enzyme, it is possible to identify possible differences in affinity and, consequently, in the metabolization rate of each of the molecules studied. LIN, an alcohol, interacts with more superficial residues, whereas LINA, an ester, interacts with deeper residues of the enzyme. The binding energies obtained by AutoDock Vina, as well as the number of hydrogen bonds formed, support this interpretation. This ester showed the lowest binding energy; then, it binds more strongly to CYP2B11 than LIN due to its higher affinity and increased hydrogen bonding. Consequently, the docking of this molecule to CYP2B11 is more stable, facilitating easier recognition of LINA as a substrate. Binding energy reflects complex stability, with lower energy indicating stronger binding and potential therapeutic or toxic effects [39]. A high binding affinity indicates a strong ligand-enzyme interaction but does not necessarily increase metabolism; excessively high affinity can reduce the reaction rate, as described by the Michaelis-Menten equation [40], where Km inversely reflects substrate affinity, but very low Km can lead to near-irreversible binding, limiting the reaction rate [41]. The low Km generates the phenomenon of “enzyme freezing”, an enzymatic inactivation that reduces metabolic capacity, which can be time-dependent or mechanism-dependent [42,43]. Furthermore, the position of the ligand in relation to the heme iron (catalytic center) of CYP also determines the velocity and efficiency of the reaction, as this site is the oxidative site of the enzyme [44]. To contextualize the “how good” is the affinity of a substrate for the binding site, aflatoxins have a binding energy with CYP of approximately −8 kcal/mol, a value close to that found for LIN binding. This binding is considered to be strong and stable [45]. Islam et al. [46] used ritonavir as a substrate, and the binding energy values were lower than those found in our study (between −13.6 and −9.9 kcal/mol). However, the values obtained by GBVI-WSA (−13 to −15.8 kcal/mol) are as negative or more negative than those found in our study, reinforcing the plausibility and stability of the interactions observed in our study. This indicates that the molecules studied here behave similarly to the standard compounds reported in the literature.

SWISS-MODEL primarily selects templates from the AlphaFold database. As AlphaFold-generated templates do not include non-protein components and often exclude non-peptide entities, such as ligands, metal ions, and prosthetic groups, the resulting model in this study lacked the heme group that is typically present in CYP enzymes. Because of the absence of heme in the model, docking was performed on the apo-form (without any bound cofactors, ligands, or prosthetic groups, such as metal ions or heme groups) of the protein, focusing on potential ligand-binding pockets predicted on the protein surface rather than a heme-defined catalytic cavity. Although this may not fully recapitulate heme-dependent interactions, our initial approach in this study was to gain insights into ligand compatibility. This agrees with other findings on the CYP active site. Terpenoids have been shown to bind to the active site of bacterial CYP107P2 [47], whereas human CYP3A4 exhibits promiscuous binding capacity across diverse substrates [48], supporting the relevance of our observed docking poses. In our study, both linalool and linalyl acetate were localized near the putative active site, suggesting a potential interaction with the functional domain. Further refinement of the model with heme incorporation is necessary for the accurate simulation of ligand interactions involving the active site. Future studies will include heme integration and refined docking to better represent the catalytic pocket and assess ligand binding in the native holoenzyme context.

The interactions between terpenoids and CYP2C21 exhibit some differences compared to those with CYP2B11. With CYP2B11, LINA interacts with a more internalized chain of CYP. Regarding CYP2C21, this ester binds in a region as superficial as LIN. Furthermore, both interact with the protein through two hydrogen bonds, suggesting similar binding strengths. Together, these data suggest that the CYP2C21 enzyme is capable of metabolizing both LIN and LINA, although possibly with a lower capacity compared to CYP2B11. Considering the binding energy, LINA appeared to have slightly more affinity for the catalytic site of CYP2C21 than LIN. Unlike CYP2B11, binding to terpenoids occurs at identical residues, suggesting that the binding pattern occurs equivalently, whereas this pattern is different in the binding of LIN and LINA to CYP2B11. Tie et al. [49] reported that substrates can occupy multiple enzyme-binding sites in different ways. This corroborates our results, showing that ligand orientation and depth relative to the enzyme’s three-dimensional structure are important for the formation of interactions.

When evaluating the interaction between LIN and CYP2D15, we observed four hydrogen bonds with the ASN 85.A and SER 62.A residues, relatively stable docking pose for this alcohol. LINA forms three hydrogen bonds with the TYR 200.A, and its interaction appears to rely more on weaker interactions, such as hydrophobic interactions or Van der Waals forces. Unlike the other CYPs evaluated, LIN, therefore, presents greater binding stability with CYP2D15 than LINA despite their similar binding energies. Near the binding region of LINA, there is an electronegative region that favors docking; however, compared to the other interactions under analysis, this region appears to be further from the binding zone, suggesting a lower affinity of the molecule for CYP2D15. Similar findings were reported by Jeong et al. [47] when studying other terpenoids in bacteria, showing that the specific positioning of the ligand is one of the determinants of the interaction; that is, deeper binding generally implies greater reactivity. Nevertheless, given the absence of heme in our models, these interpretations remain speculative and require future refinement.

Data from molecular docking suggests LINA has a slightly higher affinity for CYP enzymes than LIN. LINA shows the highest affinity for CYP2C21, while LIN has the lowest for CYP2B11. When comparing the number of hydrogen bonds, LIN had the highest number of hydrogen bonds with CYP2D15, suggesting that the LIN-CYP2D15 complex was the most stable among those evaluated. The LINA-CYP2C21 complex exhibited more favorable interactions, characterized by strong polar interactions and affinity. This indicates that hydrogen bond number alone is not predictive of overall affinity, as illustrated by the LIN–CYP2D15 interaction. CYP enzymes contain a heme group with an iron (Fe^2+^) ion at the center, serving as the catalytic site. This catalytic site exhibits a hydrophobic characteristic, which facilitates its interaction with xenobiotics, especially those with a more nonpolar character [50,51]. Some molecules, such as terpenoids, steroids, and polycyclic aromatic compounds, position themselves in a highly precise manner in relation to heme, favoring substrate-enzyme interactions [52,53], demonstrating how proximity to iron is related to ligand recognition. The metabolizing potential of CYP depends on its affinity for the substrate, its binding strength, and the nature of the residues, which, therefore, define the stability of the complex. As mentioned previously, substrate-enzyme binding alone is insufficient for metabolism. The substrate-enzyme interaction must be ideal for catalysis; the position of the substrate, binding energy, and the binding region determine whether biotransformation will occur [25,54]. When examining the effect on metabolism, more stable complexes will enhance metabolic efficiency only if the substrate is favorably positioned relative to the catalytic center (heme iron). Therefore, while LINA exhibited favorable polar interactions, the orientation of LIN within CYP2D15 may represent a more catalytically significant binding mode than that of LINA. This highlights the importance of prioritizing spatial positioning over merely counting bonds when predicting metabolic outcomes.

The present study also analyzed the pharmacophoric properties of LIN and LINA. Knowledge of the pharmacophoric features of a ligand is essential for predicting how and where it may interact with a target protein. The number and diversity of these features often reflect a ligand’s potential to bind with multiple regions of the active site, influencing both its binding affinity and specificity [55]. Generally, a greater number of pharmacophoric features corresponds to a greater potential for stabilizing interactions. For LIN, five pharmacophoric features were identified, whereas LINA exhibited seven, suggesting that the ester form may have enhanced capacity to interact with diverse regions within the enzyme’s active site. LIN contains two hydrophobic regions, whereas LINA contains five, which may favor its binding to the predominantly nonpolar environment near the heme group of CYP enzymes [55]. Although LIN possesses a dual region capable of both donating and accepting hydrogen bonds, LINA features a region limited to hydrogen-bond acceptance, indicating different interaction dynamics with the binding site that can affect complex stability.

Additionally, LIN has two AtomQ regions (associated with non-classical interactions such as van der Waals forces), while LINA has only one. Although these interactions are weaker, they can still contribute to the overall stability and specificity of ligand-enzyme complexes because they influence the positioning and orientation of the ligand at the active site. Some compounds have ideal pharmacophoric features for interaction with CYP enzymes. For example, anticancer drugs exhibit van der Waals interactions with the heme group and proximal residues, which significantly contribute to binding stability, despite variations in the number of strong bonds [56]. The contribution of GLN and TYR residues is noteworthy, since both are frequently implicated in stabilizing CYP–ligand complexes through hydrogen bonding [55,57]. Together, these pharmacophoric differences suggest distinct binding behaviors between the two molecules rather than a prediction of metabolic susceptibility. The metabolic potential of a compound is determined not only by its pharmacophoric properties but also by its orientation and proximity to the catalytic site [55]. In the present study, pharmacophoric analysis was integrated with molecular docking to provide complementary insights into binding behavior, recognizing that further validation through molecular dynamics and in vitro assays will be required.

Molecular docking analysis revealed that LIN exhibits a better binding affinity with CYP2B11, characterized by a more favorable docking score and a lower GBVI/WSA energy. The more negative the value, the stronger and more stable the binding becomes [58]. The two hydrogen bonds formed with the GLN and ASP residues (polar) contributed to greater structural stability at the binding site, in addition to favoring better and firmer positioning of the molecule within the protein, compared to the single hydrogen bond formed between LINA and the HIS residue (slightly polar), suggesting a weaker and less specific interaction [59]. These findings reinforce that LIN is more likely than LINA to form stable and catalytically relevant interactions with CYP2B11.

Upon comparing the molecular docking results of the three CYPs and the terpenoids, LIN was found to have better affinity and more specific interactions in general, with a particular emphasis on CYP2B11 and CYP2C21. This may occur due to the lower molecular weight of LIN, its greater polarity and flexibility, and chemical grouping (alcohol), which facilitates docking and the formation of more interactions [60]. The increase in nonpolar character, rigidity, and molecular weight of LINA, which is an ester, makes its docking and interactions less efficient and specific, especially with CYP2D15 [60]. Thus, LIN exhibited greater binding stability to the studied CYPs. These findings are consistent with previous reports on monoterpenes and CYP enzymes. A study conducted with the monoterpene α-terpinyl acetate, a compound present in several essential oils, demonstrated that this substance strongly binds to the human isoform CYP2B6, the ortholog of canine CYP2B11. Furthermore, molecular docking analyses confirmed the direct interaction of this terpene with the active site of the enzyme [61]. Although this study was conducted in humans, it suggests that structurally similar compounds, such as linalool, may exhibit comparable metabolic behaviors and binding patterns in dogs. In rodent studies, exposure to monoterpenes such as camphor, menthol, and α-pinene resulted in significant induction of CYP2B subtype enzymes [62]. Because the CYP2B subfamily in rodents is functionally analogous to CYP2B11 in dogs, these data indicate that terpenoid compounds have the potential to modulate the activity and expression of CYP enzymes in mammals, including in vivo studies. These findings support the plausibility of the results of this study, suggesting that LIN, owing to its similar structure, may also interact with and be preferentially metabolized by isoforms such as CYP2B11 and CYP2C21 in dogs compared to bulkier and less polar compounds such as linalyl acetate.

For comparison, Lee et al. [61] reported that terpenoids, such as α-terpinyl acetate, exhibit stable binding to CYP2B6, the human ortholog of canine CYP2B11, highlighting the structural compatibility of monoterpenes with CYP catalytic pockets. Similarly, Kramlinger et al. [63] showed that menthol and menthofuran bind with high affinity to CYP2A isoforms, acting as competitive inhibitors of nicotine oxidation. These examples illustrate that the binding of essential oil constituents to CYP enzymes is a consistent and biologically relevant phenomenon, supporting the plausibility of our observations with linalool and linalyl acetate.

The presented findings indicate that ligands with a higher affinity for CYP enzymes and increased interaction potential, such as LIN, are more likely to undergo metabolic transformation. This study found that the CYP2B11 and CYP2C21 enzymes exhibited a higher ability to interact with LIN, suggesting their potential role in the biotransformation of this monoterpene. In silico investigations of various monoterpenes have revealed similar patterns, where computational predictions of affinity were later corroborated by in vitro metabolic assays, highlighting the translational importance of these techniques [29,30,38,47,61,62,63]. These results are predictive and act as a preliminary stage for in vitro studies that can evaluate metabolic depletion and drug interaction profiles at a cellular level. The in silico approach has limitations, including differences between modeled and native enzymes, treating proteins as rigid in docking simulations, and calculated binding energies that do not fully represent cellular or physiological complexity. Future research may involve molecular modeling of CYP3A12, the major hepatic enzyme in dogs, as well as docking analyses focused on ligand positioning relative to the heme iron to better predict metabolic relevance. Furthermore, in silico screening of potential enzyme inducers or inhibitors can improve the prediction of drug interaction risks.

## 4. Materials and Methods

### 4.1. Molecular Modeling of the Target Proteins

The three-dimensional structures of canine CYP2B11, CYP2C21, and CYP2D15 were predicted using the SWISS-MODEL homology modeling server [64]. Protein sequences in FASTA format were retrieved from the NCBI Protein database under accession numbers NP_001006653.1, NP_001183973.1, and BAA20357.1, respectively. Models were generated by aligning each sequence with templates from the AlphaFoldDB, selected automatically based on sequence identity, alignment coverage, and structural quality. Model validation included assessment of Global Model Quality Estimation (GMQE) and QMEAN Z-score values. The final models were exported in PDB format for subsequent molecular docking analyses with linalool and linalyl acetate.

### 4.2. Structure-Based Drug Designing—Preparation of Proteins and Ligand Compound Molecules for Docking Analysis

Following the generation of CYP2B11, CYP2C21, and CYP2D15 3D structures, protein preparation was performed using the MGLTools suite (v1.5.7) and AutoDock Tools (ADT) [65]. Water molecules, ligands, and cofactors were removed; polar hydrogens were added, and Kollman partial charges were assigned. Structural inspection and topology verification were carried out in ADT. The docking grid was defined to encompass the full protein volume, excluding the heme group. Proteins were saved in .pdbqt format and imported into AutoDock Vina (v1.1.2) for docking.

Ligands (linalool and linalyl acetate) were retrieved from the PubChem database in .mol or .sdf formats, converted to .pdb using OpenBabel (v3.1.1) [66], and optimized by adding hydrogen atoms and generating 3D conformations. Ligand preparation in ADT involved assigning Gasteiger charges and identifying rotatable bonds. Docking was performed using default Vina parameters with rigid receptors and flexible ligands. Resulting complexes were visualized in UCSF Chimera [67] to identify hydrogen bonds and interacting residues.

### 4.3. Ligand-Based Drug Design

#### 4.3.1. Molecular Docking

Pharmacophore modeling and docking refinement of linalool and linalyl acetate were conducted using the Molecular Operating Environment (MOE) software (Chemical Computing Group, Montreal, QC, Canada). MOE supports structure- and ligand-based drug design, including modules for virtual screening, interaction prediction, and molecular visualization. LigPlot, integrated within MOE, was employed to generate 2D diagrams of protein–ligand interactions, enabling identification of key residues involved in hydrogen bonding and hydrophobic contacts, as well as visualization of interaction geometries.

Docking simulations were performed in MOE to refine the binding conformations of the ligands with CYP2B11, CYP2C21, and CYP2D15. The lowest-energy poses were selected for further analysis. Post-docking evaluation included identification of hydrogen bonds and π–π interactions, along with root mean square deviation (RMSD) calculations to assess conformational stability and spatial overlap between predicted and reference structures. Low RMSD values were interpreted as indicative of reliable docking conformations [68,69].

#### 4.3.2. Ligands Preparation

Ligand structures for CYP2B11, CYP2C21, and CYP2D15 were constructed using the MOE Builder application (Chemical Computing Group) [70]. The molecular structures of linalool (LIN) and linalyl acetate (LINAct) were imported into MOE in .mol or .sdf format. Energy minimization was carried out using the MMFF94X force field with a gradient threshold of 0.05 and chiral constraints set to the current geometry. Explicit hydrogen atoms were added, and stereochemistry corrections were applied when necessary.

Key physicochemical properties, including molecular weight, logP, polar surface area (PSA), and the number of hydrogen bond donors and acceptors, were evaluated to support the characterization of the ligands. Subsequently, the MOE Pharmacophore Query Editor was employed to identify and visualize the primary pharmacophoric features, including hydrogen bond donor and acceptor groups, hydrophobic regions, aromatic rings, and charged moieties. The resulting pharmacophore models were exported for future virtual screening and comparative structural analyses.

All energy-minimized ligands were saved in .mdb format and used as input files for molecular docking using the MOE-Dock protocol [71,72].

#### 4.3.3. Protein Preparation

Three-dimensional protonation and preparation of the target proteins CYP2B11, CYP2C21, and CYP2D15 were performed using MOE (Chemical Computing Group). Protein structures were imported in .pdb format, and energy minimization was carried out using the MMFF94X force field with solvation parameters enabled. The minimization protocol employed a gradient threshold of 0.05 and chiral constraints set to the current geometry, terminating once the root mean square (RMS) gradient fell below 0.05. The resulting energy-minimized protein structures were used as docking templates in subsequent modeling steps [71].

## 5. Conclusions

The present study predicted the molecular interactions of linalool (LIN) and linalyl acetate (LINAct) with canine CYP2B11, CYP2C21, and CYP2D15 using in silico methods. Linalool exhibited greater overall affinity, particularly for CYP2B11, forming stable interactions, such as hydrogen bonds. Limitations include the absence of heme-dependent interactions, the lack of molecular dynamics simulations, and potential differences between the modeled and native enzymes. Future work will involve in vitro validation, docking with additional enzymes (e.g., CYP3A12), and incorporation of the heme group to better represent the interactions at the catalytic site.

## Figures and Tables

**Table 1:** Molecular docking binding energy of ligand compounds linalool and linalyl acetate with interacting residues of proteins CYP2B11, CYP2C21, and CYP2D15.

Protein	Compound Ligand Name	Autodock Vina Binding Affinity	Binding Residues/Number of Hydrogen Bonds
CYP2B11	Linalool	−4.096	LYS 122, ARG 126/2
CYP2B11	Linalyl Acetate	−4.643	ASN 397, LYS 479/3
CYP2C21	Linalool	−4.487	ASN 474, SER 51/2
CYP2C21	Linalyl Acetate	−4.998	ASN 474, SER 51/2
CYP2D15	Linalool	−4.144	SER 62, GLN 65, ASN 85 and ASN 401/4
CYP2D15	Linalyl Acetate	−4.199	ARG 197 and TYR 200/3

**Table 2:** Molecular docking binding score and energy of ligand compounds linalool and linalyl acetate with proteins CYP2B11, CYP2C21, CYP2D15, and CYP2B11.

Protein	Compound Ligand Name	Docking Scores	GBVI/WSA ^1^	Binding Residues/Number of Hydrogen Bonds
CYP2B11	Linalool	−7.624	−15.8441	GLN 59 and ASP 64/2
CYP2B11	Linalyl Acetate	−6.917	−13.7862	HIS 400/1
CYP2C21	Linalool	−7.612	−13.345	ASN 204, SER 208/2
CYP2C21	Linalyl Acetate	−6.983	−12.722	No interaction
CYP2D15	Linalool	−6.533	−13.6314	HIS 112/1
CYP2D15	Linalyl Acetate	−6.711	−13.2242	ASN 255/1

^1^ The Generalized-Born Volume Integral/Weighted Surface Area (GBVI/WSA) is a scoring function that estimates the free energy of binding ligand from a given pose. For all scoring functions, lower scores indicate more favorable poses. In molecular docking studies, binding energies and docking scores are the primary bases for the selection of active and non-active compounds.

## Data Availability

Data is contained within the article.

## Abbreviations

The following abbreviations are used in this manuscript:

3D	Three-dimensional
ADMET	Absorption, distribution, metabolism, excretion, and toxicity
ADT	AutoDock Tools
ARG	Arginine
ASN	Asparagine
ASP	Aspartic acid
AtomQ	Qualifying Atom
CYP450	Cytochrome P450
DonAcc	Dual donor/acceptor
F	Feature number
GBVI/WSA	Generalized-born volume integral/weighted surface area
GLN	Glutamine
GMQE	Global model quality estimate
Hyd	Hydrophobic region
ILE	Isoleucine
K_m_	Michaelis-Menten constant
LEU	Leucine
LIN	Linalool
LINAct	Linalyl acetate
MOE	Molecular Operating Environment
PHE	Phenylalanine
PSA	Polar surface area
RMS	Root mean square
RMSD	Root mean square deviation
SER	Serine
THR	Threonine
TYR	Tyrosine
VAL	Valine

## References

[1] Chen J., Lin A., Luo P. (2024). Advancing pharmaceutical research: A comprehensive review of cutting-edge tools and technologies. Curr. Pharm. Anal..

[2] Wooller S.K., Benstead-Hume G., Chen X., Ali Y., Pearl F.M.G. (2017). Bioinformatics in translational drug discovery. Biosci. Rep..

[3] Niazi S.K., Mariam Z. (2023). Computer-aided drug design and drug discovery: A prospective analysis. Pharmaceuticals.

[4] Oselusi S.O., Dube P., Odugbemi A.I., Akinyede K.A., Ilori T.L., Egieyeh E., Sibuyi N.R., Meyer M., Madiehe A.M., Wyckoff G.J. (2024). The role and potential of computer-aided drug discovery strategies in the discovery of novel antimicrobials. Comput. Biol. Med..

[5] Adelusi T.I., Oyedele A.-Q.K., Boyenle I.D., Ogunlana A.T., Adeyemi R.O., Ukachi C.D., Idris M.O., Olaoba O.T., Adedotun I.O., Kolawole O.E. (2022). Molecular modeling in drug discovery. Inform. Med. Unlocked.

[6] Bibow A., Oleszek W. (2024). Essential oils as potential natural antioxidants, antimicrobial, and antifungal agents in active food packaging. Antibiotics.

[7] Crișan I., Ona A., Vârban D., Muntean L., Vârban R., Stoie A., Mihăiescu T., Morea A. (2023). Current trends for Lavender (Lavandula angustifolia Mill.) crops and products with emphasis on essential oil quality. Plants.

[8] Angulo S.M., Occhieppo V.B., Moya C., Crespo R., Bregonzio C. (2025). Anxiolytic-like effect characterization of essential oil from local lavender cultivation. Pharmaceuticals.

[9] Genovese A.G., McLean M.K., Khan S.A. (2012). Adverse reactions from essential oil-containing natural flea products exempted from Environmental Protection Agency regulations in dogs and cats. J. Vet. Emerg. Crit. Care.

[10] Gans J.H., Korson R., Cater M.R., Ackerly C.C. (1980). Effects of short-term and long-term theobromine administration to male dogs. Toxicol. Appl. Pharmacol..

[11] Villar D., Knight M.J., Hansen S.R., Buck W.B. (1994). Toxicity of melaleuca oil and related essential oils applied topically on dogs and cats. Vet. Hum. Toxicol..

[12] Khan S.A., McLean M.K., Slater M.R. (2014). Concentrated tea tree oil toxicosis in dogs and cats: 443 cases (2002–2012). J. Am. Vet. Med. Assoc..

[13] Sudekum M., Poppenga R.H., Raju N., Braselton W.E. (1992). Pennyroyal oil toxicosis in a dog. J. Am. Vet. Med. Assoc..

[14] Ebani V.V., Mancianti F. (2020). Use of essential oils in veterinary medicine to combat bacterial and fungal infections. Vet. Sci..

[15] Chen L., Li Q., Nasif K.F.A., Xie Y., Deng B., Niu S., Pouriyeh S., Dai Z., Chen J., Xie C.Y. (2024). AI-driven deep learning techniques in protein structure prediction. Int. J. Mol. Sci..

[16] Lohning A.E., Levonis S.M., Williams-Noonan B., Schweiker S.S. (2017). A practical guide to molecular docking and homology modelling for medicinal chemists. Curr. Top. Med. Chem..

[17] Pinzi L., Rastelli G. (2019). Molecular docking: Shifting paradigms in drug discovery. Int. J. Mol. Sci..

[18] Muegge I., Mukherjee P. (2016). An overview of molecular fingerprint similarity search in virtual screening. Expert Opin. Drug Discov..

[19] Muhammed M.T., Aki-Yalcin E. (2019). Homology modeling in drug discovery: Overview, current applications, and future perspectives. Chem. Biol. Drug Des..

[20] Zawaira A., Ching L.Y., Coulson L., Blackburn J., Wei Y.C. (2011). An expanded, unified substrate recognition site map for mammalian cytochrome P450s: Analysis of molecular interactions between 15 mammalian CYP450 isoforms and 868 substrates. Curr. Drug Metab..

[21] Martiny V.Y., Carbonell P., Chevillard F., Moroy G., Nicot A.B., Vayer P., Villoutreix B.O., Miteva M.A. (2015). Integrated structure- and ligand-based in silico approach to predict inhibition of cytochrome P450 2D6. Bioinformatics.

[22] Mestres J. (2005). Structure conservation in cytochromes P450. Proteins.

[23] Szklarz G.D., Graham S.E., Paulsen M.D. (2000). Molecular modeling of mammalian cytochromes P450: Application to study enzyme function. Vitam. Horm..

[24] Chothia C., Lesk A.M. (1986). The relation between the divergence of sequence and structure in proteins. EMBO J..

[25] Werck-Reichhart D., Feyereisen R. (2000). Cytochromes P450: A success story. Genome Biol..

[26] Laskowski R.A., MacArthur M.W., Moss D.S., Thornton J.M. (1993). PROCHECK: A program to check the stereochemical quality of protein structures. J. Appl. Crystallogr..

[27] Ayinla Z.A., Ademakinwa A.N., Agboola F.K. (2023). Comparative modelling, molecular docking and immobilization studies on Rhizopus oryzae lipase: Evaluation of potentials for fatty acid methyl esters synthesis. J. Biomol. Struct. Dyn..

[28] Binbay F.A., Rathod D.C., George A.A.P., Imhof D. (2023). Quality Assessment of selected protein structures derived from homology modeling and AlphaFold. Pharmaceuticals.

[29] Chadha A., Madyastha K.M. (1982). Omega-hydroxylation of acyclic monoterpene alcohols by rat lung microsomes. Biochem. Biophys. Res. Commun..

[30] Chadha A., Madyastha K.M. (1984). Metabolism of geraniol and linalool in the rat and effects on liver and lung microsomal enzymes. Xenobiotica.

[31] Meesters R.J., Duisken M., Hollender J. (2007). Study on the cytochrome P450-mediated oxidative metabolism of the terpene alcohol linalool: Indication of biological epoxidation. Xenobiotica.

[32] Ferreira L.G., Dos Santos R.N., Oliva G., Andricopulo A.D. (2015). Molecular docking and structure-based drug design strategies. Molecules.

[33] Schneider H.-J., Böhm H.-J., Schneider G. (2003). Introduction to molecular recognition models. Protein-Ligand Interactions: From Molecular Recognition to Drug Design.

[34] IUPAC-IUB Joint Commission on Biochemical Nomenclature (JCBN) (1984). Nomenclature and symbolism for amino acids and peptides Recommendations 1983. Biochem. J..

[35] Kumar S., Kumar S., Roy K. (2019). Molecular docking: A structure-based approach for drug repurposing. Silico Drug Design.

[36] Miyazawa M., Sugie A., Shimada T. (2003). Roles of human CYP2A6 and 2B6 and rat CYP2C11 and 2B1 in the 10-hydroxylation of (-)-verbenone by liver microsomes. Drug Metab. Dispos..

[37] Miyazawa M., Gyoubu K. (2007). Metabolism of (-)-fenchone by CYP2A6 and CYP2B6 in human liver microsomes. Xenobiotica.

[38] Seo K.A., Kim H., Ku H.Y., Ahn H.J., Park S.J., Bae S.K., Shin J.G., Liu K.H. (2008). The monoterpenoids citral and geraniol are moderate inhibitors of CYP2B6 hydroxylase activity. Chem. Biol. Interact..

[39] Krihariyani D., Woelansari E.D., Haryanto E., Sasongkowati R., Handayati A., Astuti S.S.E. (2024). In silico study of the potential of Brazilein sappan wood as a beta-lactamase inhibitor against extended-spectrum beta-lactamase-encoding genes. Malays. J. Med. Sci..

[40] Liu S., Liu S. (2017). Enzymes. Bioprocess Engineering.

[41] Shenouda J., Green P., Sultatos L. (2009). An evaluation of the inhibition of human butyrylcholinesterase and acetylcholinesterase by the organophosphate chlorpyrifos oxon. Toxicol. Appl. Pharmacol..

[42] Maurer T.S., Tabrizi-Fard M.A., Fung H.L. (2000). Impact of mechanism-based enzyme inactivation on inhibitor potency: Implications for rational drug discovery. J. Pharm. Sci..

[43] Yadav S., Mody T.A., Sharma A., Bachhawat A.K. (2020). A genetic screen to identify genes influencing the secondary redox couple NADPH/NADP^+^ in the yeast Saccharomyces cerevisiae. G3 Genes Genomes Genet..

[44] Isin E.M., Guengerich F.P. (2008). Substrte binding to cytochromes P450. Anal. Bioanal. Chem..

[45] Wu J., Zhu S., Wu Y., Jiang T., Wang L., Jiang J., Wen J., Deng Y. (2019). Multiple CH/π interactions maintain the binding of aflatoxin B_1_ in the active cavity of human cytochrome P450 1A2. Toxins.

[46] Islam M.A., Dudekula D.B., Rallabandi V.P.S., Srinivasan S., Natarajan S., Chung H., Park J. (2022). Identification of potential cytochrome P450 3A5 inhibitors: An extensive virtual screening through molecular docking, negative image-based screening, machine learning and molecular dynamics simulation studies. Int. J. Mol. Sci..

[47] Jeong E., Kim V., Kim C., Lee Y.B., Kim D. (2024). Structural insights into the interaction of terpenoids with Streptomyces avermitilis CYP107P2. Biomol. Ther..

[48] Ohkura K., Kawaguchi Y., Watanabe Y., Masubuchi Y., Shinohara Y., Hori H. (2009). Flexible structure of cytochrome P450: Promiscuity of ligand binding in the CYP3A4 heme pocket. Anticancer Res..

[49] Tie Y., McPhail B., Hong H., Pearce B.A., Schnackenberg L.K., Ge W., Buzatu D.A., Wilkes J.G., Fuscoe J.C., Tong W. (2012). Modeling chemical interaction profiles: II. Molecular docking, spectral data-activity relationship, and structure-activity relationship models for potent and weak inhibitors of cytochrome P450 CYP3A4 isozyme. Molecules.

[50] Kirton S.B., Baxter C.A., Sutcliffe M.J. (2002). Comparative modelling of cytochromes P450. Adv. Drug Deliv. Rev..

[51] Guengerich F.P. (2008). Cytochrome p450 and chemical toxicology. Chem. Res. Toxicol..

[52] Cupp-Vickery J., Anderson R., Hatziris Z. (2000). Crystal structures of ligand complexes of P450eryF exhibiting homotropic cooperativity. Proc. Natl. Acad. Sci. USA.

[53] Mast N., White M.A., Bjorkhem I., Johnson E.F., Stout C.D., Pikuleva I.A. (2008). Crystal structures of substrate-bound and substrate-free cytochrome P450 46A1, the principal cholesterol hydroxylase in the brain. Proc. Natl. Acad. Sci. USA.

[54] Nair P.C., McKinnon R.A., Miners J.O. (2016). Cytochrome P450 structure-function: Insights from molecular dynamics simulations. Drug Metab. Rev..

[55] Gay S.C., Roberts A.G., Halpert J.R. (2010). Structural features of cytochromes P450 and ligands that affect drug metabolism as revealed by X-ray crystallography and NMR. Future Med. Chem..

[56] Panneerselvam S., Yesudhas D., Durai P., Anwar M.A., Gosu V., Choi S. (2015). A combined molecular docking/dynamics approach to probe the binding mode of cancer drugs with cytochrome P450 3A4. Molecules.

[57] Sarkhel S., Desiraju G.R. (2004). N-H…O, O-H…O, and C-H…O hydrogen bonds in protein-ligand complexes: Strong and weak interactions in molecular recognition. Proteins.

[58] Labute P. (2008). The generalized Born/volume integral implicit solvent model: Estimation of the free energy of hydration using London dispersion instead of atomic surface area. J. Comput. Chem..

[59] Chan A.W., Laskowski R.A., Selwood D.L. (2010). Chemical fragments that hydrogen bond to Asp, Glu, Arg, and His side chains in protein binding sites. J. Med. Chem..

[60] Nguyen D.D., Xiao T., Wang M., Wei G.W. (2017). Rigidity strengthening: A mechanism for protein-ligand binding. J. Chem. Inf. Model..

[61] Lee Y., Park H.G., Kim V., Cho M.A., Kim H., Ho T.H., Cho K.S., Lee I.S., Kim D. (2018). Inhibitory effect of α-terpinyl acetate on cytochrome P450 2B6 enzymatic activity. Chem. Biol. Interact.

[62] Austin C.A., Shephard E.A., Pike S.F., Rabin B.R., Phillips I.R. (1988). The effect of terpenoid compounds on cytochrome P-450 levels in rat liver. Biochem. Pharmacol..

[63] Kramlinger V.M., von Weymarn L.B., Murphy S.E. (2012). Inhibition and inactivation of cytochrome P450 2A6 and cytochrome P450 2A13 by menthofuran, β-nicotyrine and menthol. Chem. Biol. Interact.

[64] Waterhouse A., Bertoni M., Bienert S., Studer G., Tauriello G., Gumienny R., Heer F.T., de Beer T.A.P., Rempfer C., Bordoli L. (2018). SWISS-MODEL: Homology modelling of protein structures and complexes. Nucleic Acids Res..

[65] Morris G.M., Huey R., Lindstrom W., Sanner M.F., Belew R.K., Goodsell D.S., Olson A.J. (2009). AutoDock4 and AutoDockTools4: Automated docking with selective receptor flexibility. J. Comput. Chem..

[66] O’Boyle N.M., Banck M., James C.A., Morley C., Vandermeersch T., Hutchison G.R. (2011). Open Babel: An open chemical toolbox. J. Cheminform..

[67] Pettersen E.F., Goddard T.D., Huang C.C., Couch G.S., Greenblatt D.M., Meng E.C., Ferrin T.E. (2004). UCSF Chimera-a visualization system for exploratory research and analysis. J. Comput. Chem..

[68] Kitchen D.B., Decornez H., Furr J.R., Bajorath J. (2004). Docking and scoring in virtual screening for drug discovery: Methods and applications. Nat. Rev. Drug Discov..

[69] Wadood A., Riaz M., Jamal S.B., Shah M. (2014). Interactions of ketoamide inhibitors on HCV NS3/4A protease target: Molecular docking studies. Mol. Biol. Rep..

[70] Riasat I., Bakhtiar S.M., Faheem M., Jaiswal A.K., Naeem M., Khan R., Khan A.U., Khalil A.A.K., Haider A., Junaid M. (2022). Application of pan genomics towards the druggability of *Clostridium botulinum*. Appl. Nanosci..

[71] Wadood A., Jamal S.B., Riaz M., Mir A. (2014). Computational analysis of benzofuran-2-carboxlic acids as potent Pim-1 kinase inhibitors. Pharm. Biol..

[72] Faheem M., Jamal S.B. (2020). Identification of Zika Virus NS5 novel inhibitors through virtual screening and docking studies. Life Sci..

